# Impact of Chitosan-Based Foliar Application on the Phytochemical Content and the Antioxidant Activity in Hemp (*Cannabis sativa* L.) Inflorescences

**DOI:** 10.3390/plants12213692

**Published:** 2023-10-26

**Authors:** Romina Beleggia, Anna Iannucci, Valeria Menga, Filippo Quitadamo, Serafino Suriano, Cinzia Citti, Nicola Pecchioni, Daniela Trono

**Affiliations:** 1 Council for Agricultural Research and Economics (CREA), Research Centre for Cereal and Industrial Crops, S.S. 673, Km 25,200, 71122 Foggia, Italy; romina.beleggia@crea.gov.it (R.B.); valeria.menga@crea.gov.it (V.M.); filippoquitadamo@libero.it (F.Q.); serafino.suriano@crea.gov.it (S.S.); nicola.pecchioni@crea.gov.it (N.P.); 2 Department of Life Science, University of Modena and Reggio Emilia, Via G. Campi 103, 41125 Modena, Italy; cinzia.citti@unimore.it; 3 CNR NANOTEC-Institute of Nanotechnology, Via Monteroni, 73100 Lecce, Italy

**Keywords:** industrial hemp, inflorescences, chitosan, elicitor, phenolic compounds, cannabinoids, tocopherols, antioxidant activity

## Abstract

In the present study, the phytochemical content and the antioxidant activity in the inflorescences of the monoecious hemp cultivar Codimono grown in southern Italy were assessed, and their elicitation was induced by foliar spray application of 50 mg/L and 250 mg/L of chitosan (CHT) at three different molecular weights (low, CHT L; medium, CHT M; high CHT H). The analysis of the phytochemical profile confirmed that cannabinoids were the most abundant class (54.2%), followed by flavonoids (40.3%), tocopherols (2.2%), phenolic acids (1.9%), and carotenoids (1.4%). Cannabinoids were represented almost exclusively by cannabidiol, whereas cannabigerol and Δ^9^-tetrahydrocannabinol were detected at very low levels (the latter was below the legal limit of 0.3%). The most abundant flavonoids were orientin and vitexin, whereas tocopherols were mainly represented by α-tocopherol. The antioxidant activity was found to be positively correlated with flavonoids and tocopherols. Statistical analysis revealed that the CHT treatments significantly affected the phytochemical content and the antioxidant activity of hemp inflorescences. Notably, a significant increase in the total phenolic content (from +36% to +69%), the α-tocopherol (from +45% to +75%) and β+γ-tocopherol (from +35% to +82%) contents, and the ABTS radical scavenging activity (from +12% to +28%) was induced by all the CHT treatments. In addition, treatments with CHT 50 solutions induced an increase in the total flavonoid content (from +12% to +27%), as well as in the vitexin (from +17% to +20%) and orientin (from +20% to +30%) contents. Treatment with CHT 50 L almost always resulted in the greatest increases. Overall, our findings indicated that CHT could be used as a low-cost and environmentally safe elicitor to improve the health benefits and the economic value of hemp inflorescences, thus promoting their employment in the food, pharmaceutical, nutraceutical, and cosmetic supply chains.

## 1. Introduction

The terms ‘hemp’ and ‘industrial hemp’ refer to those cultivars of *Cannabis sativa*, which, unlike marijuana, contain very low levels (less than 0.3%) of the psychoactive cannabinoid Δ^9^-tetrahydrocannabinol (THC) [1]. Hemp cultivation started in Central Asia roughly 12,000 years ago and gradually spread worldwide [2,3], but in the mid-20th century its cultivation was prohibited in many countries, including Italy, due to its association with marijuana. However, after hemp was properly classified, and with the environmental and industrial benefits of this species being fully understood, the ban was lifted [4] and the area dedicated to hemp cultivation increased significantly [5].

From an agronomical point of view, industrial hemp is considered a low-input and low-impact crop; it is capable of growing without pesticides and herbicides, as it is noticeably resistant to fungi, rodents, and many types of weeds. Moreover, its residues can be used in organic farming for the control of soilborne pathogens, as they act as natural pesticides, miticides, and repellants [6]. Furthermore, the hemp plant tolerates stress from heavy metals and other pollutants [7], and for this reason, this crop is successfully used to remove contaminants from the soil [8].

Industrial hemp provides the raw material for a large number of industrial applications. Hemp fibers are used for textiles, eco-building, and automotive sectors [9], and to produce bioplastic [10]. Hemp cultivated for soil remediation can be safely distilled into ethanol and used as a source of biofuel [11]. Hemp seeds, rich in unsaturated fatty acids, tocopherols, proteins, antioxidants, and essential minerals [12], can be used in the food industry [13]. Sprouts from hemp seeds, with their high levels of bioactive compounds and antioxidant activity, also represent an attractive functional food [14].

Conversely, hemp inflorescences are still considered a by-product of the fiber and seed industry, despite their promising industrial application due to their rich phytochemical profile. The main phytochemicals are represented by cannabinoids and terpenes, which are concentrated in the resin produced by the glandular trichomes located on the surface of female inflorescences. As aforementioned, inflorescences of industrial hemp show very low levels of the psychoactive cannabinoid THC. By contrast, they are rich in the non-psychoactive cannabinoids, cannabidiol (CBD) and cannabigerol (CBG), which have recognized therapeutic and medicinal benefits [15,16]. For this reason, there has been growing interest from consumers in the utilization of CBD and CBG dietary supplements, and this has created an exploding industry of products obtained from inflorescences of different industrial hemp varieties. Among these, CBD- and CBG-enriched oils are the most widespread [15,16]. However, although today the industrial importance of hemp inflorescences lies almost exclusively in their cannabinoid content, it should be underlined that hemp inflorescences also contain other bioactive compounds, such as flavonoids and phenolic acids. These compounds may represent added value for hemp inflorescences, since they are known to be associated with a high number of health-promoting properties [17], and due to their antioxidant properties, are also used in the cosmetic industry as anti-aging agents [18]. In Italy, hemp is increasingly being cultivated for multi-end uses, with 40% of farmers harvesting the inflorescences for the cosmetic industry and the extraction of bioactive ingredients [5]. For this reason, the phytochemical profile of the inflorescences from hemp plants cultivated in Italy has been deeply investigated in the last years. These studies have concerned cultivars grown in the environments of northern [19,20,21] and central [22,23,24] Italy, whereas investigations have not yet been carried out in the areas of southern Italy.

Recent studies have reported the exogenous application of elicitors as a tool for the stimulation of secondary metabolite accumulation in plant tissues [25]. Elicitors tested for this purpose are molecules that normally have a role in the control of the major biochemical pathways involved in the plant response to biotic and abiotic stresses, including the biosynthesis of secondary metabolites, which are known to play a crucial role in counteracting the adverse effect of stressful conditions on plant [25]. Among these molecules, chitosan (CHT), produced by the deacetylation of chitin derivatives, is receiving a great deal of attention for its field applications as a biostimulant for plant growth and protection. Its application on strawberry plants grown in field conditions increased fruit yield and the accumulation of secondary metabolites [26]. Similarly, an increase in total phenolic compounds and antioxidant activity was observed in CHT-treated durum wheat seedlings, an improvement that alleviated the negative effects of salt stress [27]. Furthermore, CHT application on the whole vine before and after the onset of ripening increased the levels of phenolic compounds and promoted their transport from leaves to berries [28]. The beneficial effects of CHT depend on its structural features, such as the molecular weight (MW) and the degree of deacetylation [29]. Moreover, the plant response to CHT treatment varies according to the species or even the cultivar, thus making it difficult to generalize the effect of CHT on the accumulation of secondary metabolites in plants.

In light of the above and in consideration of the increasing interest in the industrial applications of hemp inflorescences, the present study was carried out with the aim of (i) evaluating the phytochemical profile and the antioxidant activity of hemp inflorescences from the monoecious cv. Codimono grown in southern Italy, and (ii) assessing the efficacy of exogenously applied CHT in increasing the accumulation of secondary metabolites and the antioxidant activity of hemp inflorescences.

## 2. Results

### 2.1. Characterization of the Phytochemical Profile and the Antioxidant Activity of the Hemp Inflorescences

**Figure 1:**
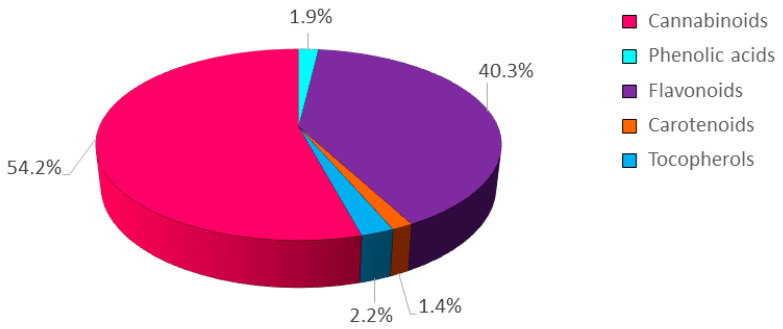
Pie chart representing the abundance of the different classes of metabolites detected in the inflorescences of the hemp cv. Codimono. Percentages were calculated from the mean values of the seven treatments.

The inflorescences of hemp plants treated with CHT solutions at two different concentrations (50 mg/L and 250 mg/L), each at three different MWs (low, L; medium, M; high, H), were analyzed for their total phenolic content (TPC), total flavonoid content (TFC), and total antioxidant activity, determined spectrophotometrically, and their content of secondary metabolites, determined by HPLC. The complete analytical data are summarized in Figure 1 and Table S1. Regarding the average abundance of the different classes of secondary metabolites detected, cannabinoids were the most represented (54.2%), followed by flavonoids (40.3%), tocopherols (2.2%), phenolic acids (1.9%), and carotenoids (1.4%) (Figure 1).

As for the individual metabolites, Codimono was characterized by very low levels of THC (below the legal limit of 0.3%) and CBG, and high levels of CBD, which accounted for about 95% of total cannabinoids (Table S1). This was expected, since this genotype belongs to chemotype III [30,31]. Phenolic acids were mainly represented by ferulic acid, which accounted for 62.3–69.4% of this class of metabolites, whereas *p*-hydroxybenzoic acid, *p*-coumaric acid and caffeic acid were less represented (11.0–16.6%, 14.5–17.3%, and 3.0–4.3%, respectively) (Table S1). The most abundant flavonoids were orientin and vitexin, which together accounted for 70.4–96.0% of total flavonoids, followed by isovitexin (12.6–18.2%), whereas epicatechin and catechin were found at low levels (1.9–3.3% and 0.9–2.6%, respectively), and luteolin and apigenin only in traces. As for the carotenoid pigments, lutein prevailed over β-carotene (59.3–64.8% and 35.2–40.7%, respectively). Within the tocopherol class, α-tocopherol was the most abundant, accounting for 86.6–89.4%, whereas β+γ-tocopherol and δ-tocopherol represented 8.1–11.2% and 1.5–2.3%, respectively.

### 2.2. Correlation Analysis of the Traits Examined in the Hemp Inflorescences

**Figure 2:**
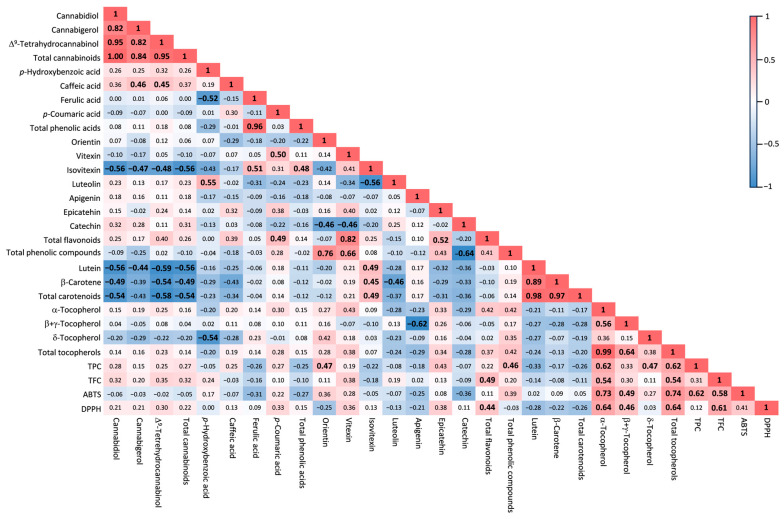
Pearson’s correlation coefficients for all pairs of traits analyzed in the inflorescences of the hemp cv. Codimono. Correlations significant at *p* ≤ 0.05 are highlighted in bold. TPC, total phenolic content; TFC, total flavonoid content; ABTS, 2,2-azinobis-(3-ethylbenzothiazoline-6-sulphonic acid) radical scavenging activity; DPPH, 2,2-diphenyl-1-picrylhydrazyl radical scavenging activity.

A correlation analysis was performed to investigate the relationship between all pairs of traits examined in the hemp inflorescences (Figure 2). As expected, significant correlations were found between metabolites in the same class. This was particularly evident for cannabinoids and carotenoids that, within their own class, were all highly correlated to each other (0.82 ≤ r ≤1.00 and *p* < 0.0001 and 0.89 ≤ r ≤1.00 and *p* < 0.0001, respectively). Interestingly, TFC was found to be positively correlated with the total flavonoids detected by HPLC (r = 0.49 and *p* = 0.023), whereas TPC had a positive correlation (r = 0.46 and *p* = 0.048) with the total phenolic compounds given by the sum of phenolic acids and flavonoids.

Regarding the correlation between metabolites belonging to different classes, there was a notably positive correlation of TPC and TFC with α-tocopherol (r = 0.62 and *p* = 0.003 and r = 0.54 and *p* = 0.011, respectively) and total tocopherols (r = 0.62 and *p* = 0.003 and r = 0.54 and *p* = 0.012, respectively), and of TPC with δ-tocopherol (r = 0.47 and *p* = 0.031). Additionally, cannabinoids were found to be negatively correlated with carotenoids (−0.59 ≤ r ≤ −0.44 and 0.005 ≤ *p* ≤ 0.048), whereas isovitexin was found to be positively correlated with carotenoids (0.45 ≤ r ≤ 0.49 and 0.025 ≤ *p* ≤ 0.040) and negatively correlated with cannabinoids (−0.56 ≤ r ≤ −0.47 and 0.009 ≤ *p* ≤ 0.032).

As far as the antioxidant activity, the 2,2-azinobis-(3-ethylbenzothiazoline-6-sulphonic acid (ABTS) and the 2,2-diphenyl-1-picrylhydrazyl (DPPH) radical scavenging activities were both positively correlated with TFC, α-tocopherol, β+γ-tocopherol, and total tocopherols (0.46 ≤ r ≤ 0.74 and 0.0001 ≤ *p* ≤ 0.037). The ABTS radical scavenging activity was also positively correlated with TPC (r = 0.62 and *p* = 0.003), whereas the DPPH radical scavenging activity was positively correlated with total flavonoids detected by HPLC (r = 0.44 and *p* = 0.044).

### 2.3. Variations in the Phytochemical Content and the Antioxidant Activity in Hemp Inflorescences Due to CHT Treatments

**Figure 3:**
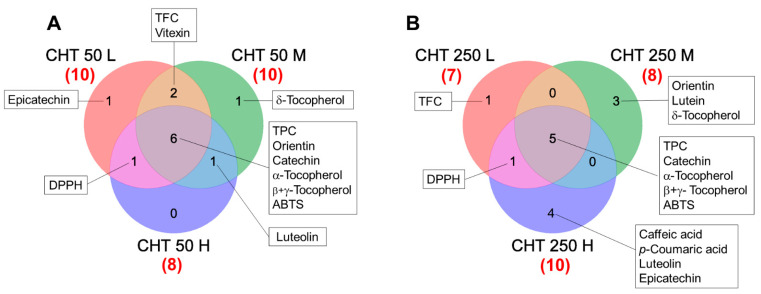
Venn diagram for differentially accumulated metabolites (DAMs) detected in the inflorescences of the hemp plants from the cv. Codimono treated with CHT solutions at 50 mg/L (**A**) and 250 mg/L concentrations (**B**). Numbers in parentheses indicate the total DAM number induced by each treatment. TPC, total phenolic content; TFC, total flavonoid content; ABTS, 2,2-azinobis-(3-ethylbenzothiazoline-6-sulphonic acid) radical scavenging activity; DPPH, 2,2-diphenyl-1-picrylhydrazyl radical scavenging activity. CHT 50 L, 50 mg/L low molecular weight chitosan; CHT 50 M, 50 mg/L medium molecular weight chitosan; CHT 50 H, 50 mg/L high molecular weight chitosan; CHT 250 L, 250 mg/L low molecular weight chitosan; CHT 250 M, 250 mg/L medium molecular weight chitosan; CHT 250 H, 250 mg/L high molecular weight chitosan.

**Figure 4:**
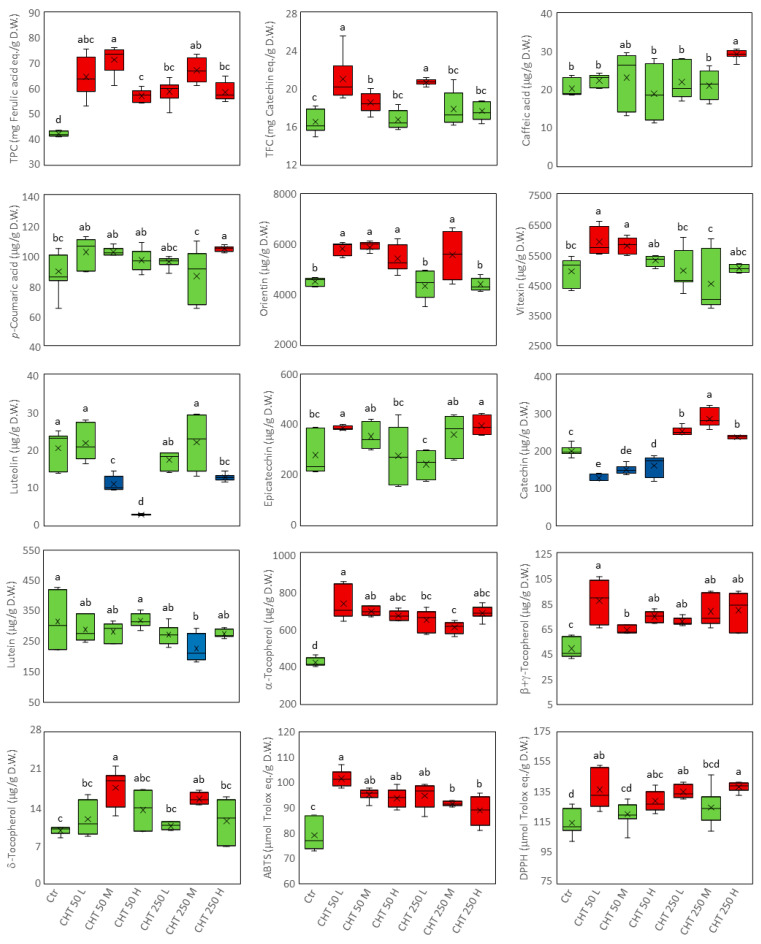
Box and whisker plots of the differentially accumulated metabolites in the inflorescences of the hemp plants from the cv. Codimono treated with CHT solutions at 50 mg/L and 250 mg/L concentrations. Boxes of metabolites that increased, decreased, or remained unchanged in CHT-treated plants compared to control plants are colored in red, blue, and green, respectively. For each metabolite, different lower-case letters represent significant differences among treatments (Tukey’s test *p* ≤ 0.05). TPC, total phenolic content; TFC, total flavonoid content; ABTS, 2,2-azinobis-(3-ethylbenzothiazoline-6-sulphonic acid) radical scavenging activity; DPPH, 2,2-diphenyl-1-picrylhydrazyl radical scavenging activity. Ctr, control; CHT 50 L, 50 mg/L low molecular weight chitosan; CHT 50 M, 50 mg/L medium molecular weight chitosan; CHT 50 H, 50 mg/L high molecular weight chitosan; CHT 250 L, 250 mg/L low molecular weight chitosan; CHT 250 M, 250 mg/L medium molecular weight chitosan; CHT 250 H, 250 mg/L high molecular weight chitosan.

To identify the metabolite variations that characterized the response of hemp inflorescences to the foliar CHT treatments, the entire dataset (Table S1) was subjected to ANOVA. Those metabolites that were differentially accumulated in at least one CHT treatment compared to the control are reported in Figure 3 and Figure 4. Seven to ten differentially accumulated metabolites (DAMs) were detected in hemp inflorescences from CHT-treated plants. Compared to the control, all the CHT treatments induced a significant increase in the TPC (from +36% to +69%), α-tocopherol (from +45% to +75%) and β+γ-tocopherol (from +35% to +82%) contents, and in the ABTS radical scavenging activity (from +12% to +28%), with the greatest increases induced by CHT 50 L. Conversely, catechin content decreased (from −20% to −37%) and increased (from +20% to +44%) under CHT 50 and CHT 250 treatments, respectively. The DPPH radical scavenging activity increased (from +12% to +21%) under all treatments except CHT 50 M and CHT 250 M. Another notable finding was the increase in the TFC (from +12% to +27%), as well as in the content of the two most abundant flavonoids, orientin (from +20% to +30%) and vitexin (from +17% to +20%), which was predominantly observed under the CHT 50 treatments. Significant variations were also observed under some CHT treatments for the content of the low-abundance compounds caffeic and *p*-coumaric acids, epicatechin, δ-tocopherol, lutein, and luteolin. It should be noted that, although not statistically significant due to the high variability among the replicates, an average decrease of 18% in the carotenoid content and an average increase of 24% in the cannabinoid content were observed in the inflorescences of plants treated with the three CHT 250 solutions (Table S1).

### 2.4. Identification of Candidate Biomarkers for CHT Treatments

**Figure 5:**
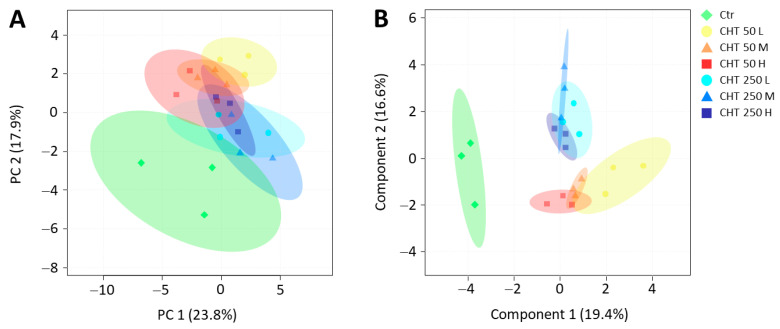
Principal component analysis (PCA) (**A**) and sparse partial least-squares-discriminant analysis (sPLS-DA) (**B**) for the traits investigated in the inflorescences of the hemp plants from the cv. Codimono treated with CHT solutions at 50 mg/L and 250 mg/L concentration. The percentages of total variance represented by the first two components are shown in parentheses. Ctr, control; CHT 50 L, 50 mg/L low molecular weight chitosan; CHT 50 M, 50 mg/L medium molecular weight chitosan; CHT 50 H, 50 mg/L high molecular weight chitosan; CHT 250 L, 250 mg/L low molecular weight chitosan; CHT 250 M, 250 mg/L medium molecular weight chitosan; CHT 250 H, 250 mg/L high molecular weight chitosan.

To assess whether DAMs can be considered promising candidate biomarkers for CHT treatments, multivariate analyses were carried out on the dataset of hemp inflorescences from control and CHT-treated plants (Figure 5). The principal component analysis (PCA) revealed that the first five principal components (PCs) represented more than 68% of the total variability in the dataset, with PC 1 and PC 2 explaining 23.8% and 17.9%, respectively (Figure S1). On the full dataset, the PCA score plot showed an unclear separation between the seven groups, with the notable exception of a slight separation of the control from the six CHT treatments (Figure 5A). As for the sparse partial least squares-discriminant analysis (sPLS-DA), the first five components explained 62% of the total variability, with component 1 and component 2 explaining 19.4% and 16.6%, respectively (Figure S2). Unlike PCA, sPLS-DA better discriminated the control from the CHT treatments on the first component and separated the CHT 50 from CHT 250 treatments on the second component. This indicated that the variable selection in the sPLS-DA produced a robust and easy-to-interpret model, which improved the clustering of treatments (Figure 5B). No clear separation was observed between the different MWs within each CHT concentration, except for the CHT 50 L treatment, which was slightly separated on the first component from the other two MWs at the same CHT concentration.

**Figure 6:**
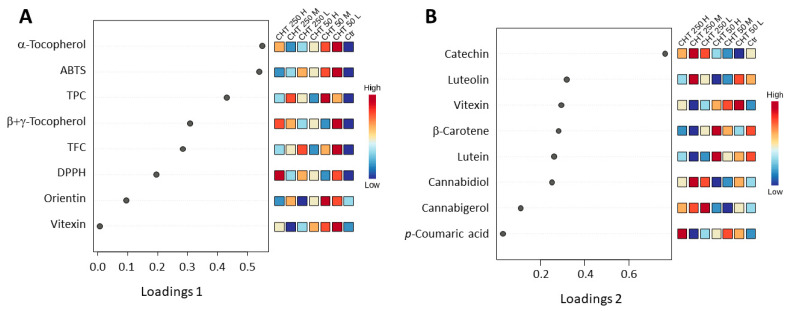
Loading plots of the eight traits that are most significant in the group separation among the different treatments for component 1 (**A**) and component 2 (**B**) of the sPLS-DA. TPC, total phenolic content; TFC, total flavonoid content; ABTS, 2,2-azinobis-(3-ethylbenzothiazoline-6-sulphonic acid) radical scavenging activity; DPPH, 2,2-diphenyl-1-picrylhydrazyl radical scavenging activity. Ctr, control; CHT 50 L, 50 mg/L low molecular weight chitosan; CHT 50 M, 50 mg/L medium molecular weight chitosan; CHT 50 H, 50 mg/L high molecular weight chitosan; CHT 250 L, 250 mg/L low molecular weight chitosan; CHT 250 M, 250 mg/L medium molecular weight chitosan; CHT 250 H, 250 mg/L high molecular weight chitosan.

Loading plots were also used to reveal which variables were most responsible for the separation observed in the sPLS-DA score plot (Figure 6). As shown in Figure 6A, the eight traits selected by the sPLS-DA model on the first component were those that were significantly induced by CHT treatments. In particular, the TPC, the α-tocopherol and β+γ-tocopherol contents, and the ABTS radical scavenging activity, which were significantly induced by all the CHT treatments (see Figure 4), ranked in the first four positions, thus revealing that these traits were the main traits responsible for the separation of the control group from the CHT-treated groups on the first component. A separation of the CHT 50 L from the other treatments was also observed, due to the highest levels detected in this treatment for the eight traits selected on the first component.

Regarding the traits selected by the sPLS-DA model on the second component (Figure 6B), which separated the CHT 50 from the CHT 250 treatments, five of the eight metabolites were significantly different at the ANOVA analysis, namely catechin, luteolin, vitexin, lutein, and *p*-coumaric acid, which corresponded to those metabolites that were affected differently by CHT 50 and CHT 250 treatments (see Figure 4). However, as said above (see Section 2.3), CHT 50 and CHT 250 treatments also differed, although not significantly, for the content of the other three metabolites, lutein, CBD, and CBG, which compared to the control condition remained almost unchanged in the CHT 50 treatments and varied in the CHT 250 treatments (see Table S1).

## 3. Discussion

### 3.1. Phytochemicals and Antioxidant Activity in the Inflorescences of the Hemp cv. Codimono

The analysis of the phytochemical profile of the inflorescences of the hemp cv. Codimono confirmed that cannabinoids represent the most abundant class of phytochemicals in this organ. As expected from a genotype belonging to chemotype III, CBD was found to be the most abundant cannabinoid [30,31]. The presence of high levels of CBD in the hemp inflorescences is important from a pharmacological/nutraceutical point of view. Indeed, although to date only one CBD product has been approved as a drug to treat seizures caused by certain conditions, the CBD product industry is experiencing an incredible growth due to the substantial interest of consumers, as well as the medical and scientific communities, due to the potential therapeutic properties of CBD that range from neuroprotective to anti-inflammatory, antipsychotic, analgesic, anticonvulsant, cardioprotective, antiarthritic, and anticancer effects [15].

Flavonoids were the second-most abundant class of metabolites detected in the hemp inflorescences. A similar observation has already been reported for other monoecious and dioecious cultivars [21], thus indicating that the high flavonoid richness is a general feature of hemp inflorescences. The two most abundant flavonoids detected were vitexin and orientin, which have already been reported as two of the flavonoids present at the highest levels in hemp inflorescences, and together with isovitexin, are considered the signature flavonoids of CBD-dominant chemotypes [32]. As already observed in previous studies on hemp inflorescences from different cultivars [21,33], TPC and TFC were found to be significantly correlated with the content of the total phenolic compounds and flavonoids, respectively, detected by HPLC. This indicates that the spectrophotometric assays represent adequate methods for assessing the content of these classes of metabolites in hemp inflorescences.

Interestingly, the presence in hemp inflorescences of tocopherols, mainly represented by α-tocopherol, was also detected. This class of secondary metabolites has been deeply characterized in hemp seed oil, in which tocopherols represent important constituents together with polyunsaturated fatty acids, terpenes, and carotenoids [34,35,36,37], whereas the little existing evidence on the presence of this class of compounds in the inflorescences has only been reported very recently [21,38]. Interestingly, the correlation analysis revealed that α-tocopherol, together with total tocopherols, was the metabolite most correlated with the antioxidant activity of hemp inflorescences. This is not surprising, since tocopherols are the most effective group of lipophilic antioxidants in plants [39]. However, this important contribution of tocopherols to the antioxidant activity of hemp inflorescences has only been highlighted by our research group in the present study and a previous study [21], while in all other studies carried out on hemp inflorescences, the major contribution was attributed to the phenolic component [23,40]. A correlation between the antioxidant activity and TPC, TFC, and total flavonoids detected by HPLC was also observed, which confirmed the contribution of these compounds to the antioxidant potential of hemp inflorescences. Interestingly, our results also showed that tocopherols were positively correlated with TPC and TFC. In this regard, evidence exists that phenolic compounds act synergistically with α-tocopherol through its regeneration from the oxidized form [41]. Thus, it is feasible that a synergic interaction between tocopherols and phenolic compounds occurs in hemp inflorescences, which can help boost their antioxidant potential.

Comparison between the phytochemical profile of hemp inflorescences reported in the present study with that previously assessed for the same cultivar in northern Italy in the same cropping season [21] revealed some interesting differences in terms of phytochemical composition and content. Compared to northern Italy, the inflorescences of plants grown in southern Italy showed (i) a halved THC content (−52%), (ii) a slightly lower content of CBD (−26%), which however remained at high levels (on average 16,400 μg/g D.W.), (iii) higher TPC, TFC, and phenolic acid, flavonoid, and tocopherol contents (from +50% to +80%). In addition, unlike plants grown in northern Italy, the flavonoid profile in the inflorescences of plants grown in southern Italy was dominated by orientin and vitexin, which together reached a level 154% higher in the latter compared to the former. These differences are not surprising, since the location in southern Italy used in the present study strongly differs from that of the previous study for its pedoclimatic conditions, which are among the frequently reported factors that affect the phytochemical content and composition of plants [42]. Overall, these findings suggest that, compared to northern Italy, the environmental conditions of southern Italy not only ensure high CBD levels and THC levels below the legal limit, but also favor greater accumulation of other beneficial secondary metabolites, i.e., phenolic compounds and tocopherols. Together with CBD, these compounds may contribute to increasing the health value of hemp inflorescences.

### 3.2. Effect of CHT-Based Foliar Treatments on Phytochemical Content and Antioxidant Activity in the Inflorescences from the Hemp cv. Codimono

Multivariate analysis revealed that hemp inflorescences responded metabolically to CHT treatments. Notably, the TPC, the α-tocopherol and β+γ-tocopherol contents, and the ABTS radical scavenging activity increased significantly under all the CHT treatments. Treatments with the CHT 50 solutions triggered higher increases compared to the CHT 250 solutions, with the greatest increases observed in the inflorescences of plants exposed to the CHT 50 L treatment. CHT 50 treatments also presented a specific increase in the TFC, particularly in the content of the two most abundant flavonoids, vitexin and orientin; also in this case, the highest increases were triggered by the CHT 50 L treatment. This is the first evidence of the ability of CHT-based treatments to induce the accumulation of secondary metabolites in hemp inflorescences. In fact, the only other study related to the treatment of hemp plants with CHT concerned the ability of this elicitor to enhance defense responses in hemp roots through the up-regulation of defense-related genes and the secretion of defense proteins in the root exudate [43]. Conversely, the ability of CHT-based applications to increase the levels of phenolic compounds has been reported in many other plant species, including many herb plants. An increase in TPC was observed in sweet basil [44] and lemon balm [45], whereas an increase in both TPC and TFC was detected in peppermint [46], sage [47], Greek oregano [48], and ginkgo biloba [49]. Similarly to the present study, some of these previous studies also highlighted the increased accumulation of specific phenolic compounds due to CHT treatment. CHT elicited the production of rosmarinic acid in basil and lemon balm [45], of caffeic acid and its derivatives in Cuban oregano [50], and of apigenin 6,8-di-*C*-glucoside in Greek oregano [48]. Regarding the possible mechanism underlying the accumulation of phenolic compounds induced by CHT, there is growing evidence of the ability of CHT to elicit the phenylpropanoid pathway through regulation at both the gene and protein levels. The application of CHT increased the expression and the activity of different enzymes involved in the biosynthesis of phenolic compounds in pear [51], citrus [52], and *Scrophularia striata* [53]. Additionally, the activity of the phenylalanine ammonia-lyase, the first key enzyme of the phenylpropanoid pathway in plants, was found to be elicited by CHT in soybean [54], rice [55], and apple [56]. This mechanism should be confirmed in hemp inflorescences by future studies. While the ability of CHT to elicit the accumulation of phenolic compounds in plants is well documented, scarce and contrasting findings are available on the effect of CHT on the accumulation of tocopherols. CHT treatments increased both the TPC and the α-tocopherol content in wheat grains [57], and an increase in the α-tocopherol content was detected in the leaves of CHT-treated horseradish plants, which was supported by the up-regulation of the gene encoding the tocopherol methyl transferase, an important enzyme regulating the synthesis of the four tocopherols [58]. Conversely, no significant variations were observed in the tocopherol content of tomato fruits in response to the treatment of plants with CHT [59].

The increased levels of phenolic compounds and tocopherols observed in the present study in CHT-treated plants, together with the consequent increase in the antioxidant activity, may contribute to enhancing the beneficial properties of both water and oil extracts obtained from hemp inflorescences and to improving the extraction yield of specific compounds from this organ. Phenolic compounds are the main components of hemp water extract. For their role in the antioxidant defense system, phenolic compounds have recognized protective effects against acute and chronic diseases [60]. Consistent with these findings, in vitro and ex vivo studies demonstrated that the phenolic extract from hemp inflorescences had antioxidant and anti-inflammatory activity and was effective against bacterial strains and fungal species involved in ulcerative colitis [61]. Another study highlighted the cytoprotective properties of the hemp phenolic extract on neuronal cell lines through the inhibition of the reactive oxygen species generation [62]. In addition to their use in the nutraceutical and pharmacological sectors, the phenolic-rich extracts from the inflorescences of hemp plants treated with CHT may also have potential use for insect pest management as an alternative to chemical pesticides. In this regard, water phenolic extracts from hemp inflorescences have demonstrated certain insecticidal activity against the larvae of the Indian meal moth [63], tobacco cutworm, and cabbage worm [64].

Phenolic compounds together with tocopherols are also important constituents of hemp oil extracts [65]. Their presence has been extensively investigated in oils extracted from hemp seeds, leading to evidence that these compounds contribute to both the oils’ oxidative stability [66] and their health benefits in the prevention of degenerative diseases, such as cardiovascular disease, Alzheimer’s disease, certain types of cancer, and age-related macular degeneration [12]. Oil extract from hemp inflorescences differs from that of seeds, since in the former, the most abundant tocopherol is α-tocopherol, whereas in the latter, γ-tocopherol prevails. α-Tocopherol is the most abundant form of vitamin E in human blood because it is the only one that is absorbed within the body and transferred by the liver to the other tissues, whereas the other isoforms are excreted through the intestine [67]. In light of this, the α-tocopherol-rich oil extracted from the inflorescences of the CHT-treated plants could be used as a valuable ingredient in food and nutraceutical preparations and provide important benefits for human health. The inflorescences of CHT-treated plants may be also a good source of vitexin and orientin that can be isolated, transformed into bioactive constituents, and incorporated into functional foods, nutraceutical supplements, or pharmaceuticals. Indeed, both these flavonoids have received great attention due to their numerous pharmacological properties, such as their anti-microbial, anti-inflammatory, anticancer, neuroprotective and cardioprotective effects [68,69].

Finally, evidence exists that the antioxidant properties of hemp extracts had a positive effect on skin by preventing the degradation of collagen and elastin fibers [70]. Furthermore, hemp extract was found to be an effective antioxidant able to reduce the lipid oxidation in linseed oil [71]. In light of these findings, it can be speculated that the high antioxidant potential detected in the inflorescences of CHT-treated plants makes them a valuable ingredient for use in cosmetics to counteract skin aging and in foods to improve the oxidative stability of vegetable oils.

## 4. Materials and Methods

### 4.1. Preparation of the Chitosan Solutions

Three types of commercial chitosan (CHT) at low (50–190 kDa), medium (190–310 kDa), and high (310–375 kDa) MWs with 75–85% deacetylation were purchased from Sigma-Aldrich (Saint Louis, MO, USA). For each type of CHT, two solutions at concentrations of 50 mg/L and 250 mg/L were prepared by dissolving the required amount of powder in 0.5% acetic acid. The pH of the solution was adjusted to 6.0 by using 1 M NaOH.

### 4.2. Plant Material and Growing Conditions

Seeds from the industrial hemp cv. Codimono were sown on 4 April 2020 at the experimental field of the Research Centre for Cereal and Industrial Crops of Foggia, Italy (41°28′ N, 15°32′ E). The sowing density was 60 kg/ha with an inter-row spacing of 75 cm on a large plot (5 m^2^) fertilized in pre-sowing according to common agronomical practice. Manual weed control was performed after sowing and during the trial duration.

The experiment was carried out using a complete randomized block design with three parcel replicates. Seven treatments were carried out as follows: (1) control (Ctr), 0.5% acetic acid; (2) CHT 50 L, 50 mg/L low MW chitosan; (3) CHT 50 M, 50 mg/L medium MW chitosan; (4) CHT 50 H, 50 mg/L high MW chitosan; (5) CHT 250 L, 250 mg/L low MW chitosan; (6) CHT 250 M, 250 mg/L medium MW chitosan; (7) CHT 250 H, 250 mg/L high MW chitosan. Starting from full flowering (50% of flowers open, BBCH 65) until the end of flowering (BBCH 69) [72], leaves were sprayed with the CHT solutions at ten-day intervals. At harvesting, ten inflorescences were randomly collected for each replicate and dried in a ventilated oven at 40 °C for 72 h. The inflorescences and the floral bracts were separated manually from stems and seeds using a 2 mm diameter sieve and stored for further analysis. Before the analysis, each sample was ground into powder using a planetary mill with jar balls (Pulverisette 7, Fritsch, Milan, Italy).

### 4.3. Spectrophotometric Measurements

#### 4.3.1. Determination of TPC and TFC

Samples were extracted according to Quitadamo et al. [27]. Briefly, 5 mL of a methanol:water (80:20 *v*/*v*) solution acidified with 1% HCl were added to 100 mg of the ground sample, and the suspension was vortexed and ultrasonicated for 30 min at room temperature. After centrifugation at 9000× *g* for 20 min at room temperature, the supernatant was transferred into 15 mL clean tubes and stored at −20 °C until TPC and TFC analysis.

TPC was determined using the Folin–Ciocalteu assay essentially as reported in Beleggia et al. [21]. Two hundred microliters of adequately diluted methanolic extract were added to 900 μL of Folin–Ciocalteu reagent diluted 1:10, then the solution was equilibrated for 5 min and mixed with 900 μL of 6% (*w*/*v*) sodium carbonate solution. The solution was mixed and its absorbance at 725 nm was measured after sitting for 60 min in the dark. All assays were performed in triplicate. Ferulic acid was used as the reference standard and the results were expressed as mg of ferulic acid equivalents/g of dry weight (D.W.).

TFC was determined according to Beleggia et al. [21] with minor modifications. Two hundred and fifty microliters of adequately diluted methanolic extract were mixed with 1 mL of distilled water and 80 µL of 5% (*w*/*v*) NaNO_2_. Five minutes later, 150 µL of 10% (*w*/*v*) AlCl_3_ were added to the mixture. Then, after another five minutes, 500 µL of 1 M NaOH were added and the total volume was made up to 3 mL with distilled water. The solution was mixed and its absorbance at 510 nm was measured. All assays were performed in triplicate. Catechin was used as the reference standard and the results were expressed as mg of catechin equivalents/g D.W.

#### 4.3.2. Determination of the Total Antioxidant Activity

The total antioxidant activity was determined by the ABTS and DPPH assays according to Beleggia et al. [21]. For both determinations, the same methanolic extract used for the TPC and TFC assays was used. The ABTS radical cation (ABTS^•+^) was prepared daily by the reaction of the ABTS solution (7 mM in water) with potassium persulfate (2.45 mM final concentration in water). After incubation for 16 h at room temperature in the dark, the ABTS^•+^ solution was diluted with ethanol to obtain an absorbance value of 0.8 at 734 nm. For the DPPH assay, a DPPH radical (DPPH^•^) solution having an absorbance value of 0.80 at 525 nm was prepared daily by dissolving 5 mg of DPPH in 100 mL methanol/water mixture (50:50, *v*/*v*). Measurements were carried out by adding 100 μL of the methanolic extract to 4.9 mL ABTS^•+^ or DPPH^•^ solution. The reaction mixture was incubated for 30 min in the dark and then the decolorization of the radical solution was evaluated by measuring the absorbance at 734 nm for the ABTS^•+^ and at 517 nm for the DPPH^•^. All assays were performed in triplicate. 6-Hydroxy-2,5,7,8-tetramethyl-chroman-carboxylic acid (Trolox), a water-soluble analogue of vitamin E, was used as the reference standard and the results were expressed as μmol of Trolox equivalent/g D.W.

### 4.4. HPLC Measurements

#### 4.4.1. Determination of Phenolic Compounds

Phenolic compounds were identified and quantified according to Kim et al. [73]. This determination was carried out on the same methanolic extract used for the spectrophotometric measurements. The HPLC system (Series 1290, Agilent Technologies, Waldbronn, Germany) was equipped with a degasser, a quaternary pump, an autosampler, a thermostated compart column and a diode-array detector (Agilent Technologies, Waldbronn, Germany). For separation, a Zorbax SB-C18 column 250 mm × 4.6 mm × 5 μm (Agilent, Santa Clara, CA, USA) was used. The temperature of the column oven was set at 35 °C. A gradient elution was employed with a mobile phase consisting of 1% acetic acid (solution A) and acetonitrile (solution B) as follows: 0 min = 95% A; 30 min = 85% A; 40 min = 50% A; 44 min = 0 A%; 46 min = 95% A; isocratic elution of 95% A, 46–50 min. The flow rate of the mobile phase was 1 mL/min, and the injection volume was 10 µL. Phenolic compounds were detected at 280 and 320 nm by recording spectra from 210 to 520 nm. The identification of each peak was confirmed using the retention time and absorbance spectrum of the corresponding authentic standard. The quantification of each phenolic acid and flavonoid was achieved by using the corresponding calibration curve. All assays were performed in triplicate and the results were reported as μg/g D.W.

#### 4.4.2. Determination of Carotenoids

Two hundred milligrams of each sample were extracted twice by adding 4 mL of water-saturated butan-1-ol. The suspension was stirred for 1 h and then centrifuged at 4000× *g* for 10 min at 8 °C. The two supernatants were pooled and filtered through a 0.22 mm PTFE membrane. Carotenoids were identified and quantified according to Ferioli et al. [74] using a HPLC system (Series 1100, Agilent Technologies, Waldbron, Germany) equipped with a degasser, a quaternary pump, a temperature-controlled injector, a temperature-controlled column thermostat, and a photodiode array detector (Agilent Technologies, Waldbronn, Germany). For separation, a C18 column Synergi 4 μm Hydro RP 250 mm × 4.6 mm (Phenomenex, Torrance, CA, USA) and a precolumn C18, 4.0 mm × 3.0 mm (Phenomenex, Torrance, CA, USA) were used. The temperature of the column oven was set at 30 °C. The elution was carried out in gradient mode using a mobile phase consisting of water (solution A) and a mobile phase consisting of acetone (solution B). The gradient program was as follows: from 0 to 5.1 min, 35% A; from 5.1 to 9.1 min, 35% to 10% A; from 9.1 to 11.9 min, 10% A; from 11.9 to 13.7 min, 10% to 0% A; from 13.7 to 16.8 min, 0% A; from 16.8 to 17.7 min, 0% to 35% A; from 17.7 to 23 min, 35% A as post-run. The flow rate was 0.8 mL/min, and the injection volume was 20 μL. Carotenoids were detected at 450 nm by recording spectra from 400 to 600 nm. The identification of each peak was confirmed using the retention time and absorbance spectrum of the corresponding authentic standard. The quantification of each carotenoid was achieved using the corresponding calibration curve. All assays were performed in triplicate, and the results were reported as μg/g D.W.

#### 4.4.3. Determination of Tocopherols

Tocopherols were extracted and determined according to Tsochatzis et al. [75], with some modifications. One hundred milligrams of each sample were extracted with 3 mL acetonitrile on a 10 mL conical glass flask for 30 min under magnetic stirring. The sample was then transferred in a 20 mL centrifuge tube, with the flask subsequently being washed twice with 2.5 mL of acetonitrile to ensure complete transfer of the sample into the tube. The sample was centrifuged at 3000× *g* for 15 min at 10 °C, and the supernatant was dried by a centrifugal evaporator (Jouan RC 1022, Thermo Electron Corp., American Laboratory Trading, Inc., East Lyme, CT, USA). The dried sample was redissolved in 1 mL methanol, vortexed for 30 s, and filtered through a 0.22-mm PTFE membrane (Millipore, Carrigtwohill Co., Cork, Ireland). Tocopherols were separated and quantified using the same HPLC system used for the determination of the phenolic compounds. The column temperature was set at 30 °C, and the injection volume was 20 µL. The mobile phase consisted of acetonitrile/methanol/2-propanol (40:55:5 *v*/*v*/*v*) under isocratic conditions at a flow rate of 0.8 mL/min, with a total analysis time of 30 min. Tocopherols were detected by fluorescence with excitation and emission wavelengths set at 292 and 335 nm, respectively. The quantification of each tocopherol was achieved using the calibration curve of the corresponding standard. The two isomers β- and γ-tocopherols coeluted because they had the same retention time, and for this reason the value reported was the sum of both forms. All assays were performed in triplicate, and the results were reported as μg/g D.W.

#### 4.4.4. Determination of Cannabinoids

Samples were extracted according to the protocol reported in the monograph of *Cannabis flos* of the German Pharmacopoeia [76]. Briefly, 5 mL of 96% ethanol were added to 100 mg of ground sample, and the suspension was stirred for 10 min at room temperature. After settling, the liquid fraction was transferred to a 10 mL volumetric flask, and the solid residue was extracted twice with 2 mL of 96% ethanol. The combined liquid fractions were brought up to 10 mL in volume with fresh ethanol. A 1 mL aliquot was centrifuged at 9000× *g* for 5 min at room temperature, and the supernatant was transferred into clean glass vials and stored at −20 °C until analysis. A 100 µL aliquot was diluted 1:10 with acetonitrile, and 5 µL of the solutions was injected into the HPLC system.

The chromatographic separation of cannabinoids was carried out on a Vanquish Core HPLC system (ThermoFisher Scientific, Waltham, MA, USA) equipped with a binary pump, a vacuum degasser, a thermostated autosampler (at 4 °C), a thermostated column compartment (at 30 °C), and a diode array detector. A Poroshell 120 EC-C18 (100 mm × 3.0 mm I.D., 2.7 µm) with guard (50 mm × 3.0 mm I.D., 2.7 µm) (both from Agilent Technologies, Milan, Italy) was employed as stationary phase, while a mixture of water (A) and acetonitrile (B), both with 0.1% formic acid, was used as mobile phase. A previously developed chromatographic method was applied for the separation of cannabinoids [77]. Briefly, a linear gradient from 5% to 95% B was set from 0 to 20 min, then the mobile phase was maintained at 95% B for 3 min. A washing step at 98% B was run from 23 to 30 min, and subsequently, the column was re-equilibrated with the initial conditions (5% B) for a further 6 min. The total run time was 36 min. The UV trace was recorded at all wavelengths (190–400 nm), and the chromatograms were processed, selecting the trace at 230 nm. The analyses were acquired with the Xcalibur 3.0 software and processed using Chromeleon 7 (ThermoFisher Scientific, Waltham, MA, USA). The identification of each peak was confirmed using the retention time and absorbance spectrum of the corresponding authentic standard. The quantification of each cannabinoid was carried out using the calibration curve of the corresponding standard. All assays were performed in triplicate, and the results were reported as μg/g D.W. For each cannabinoid, the total content comprised the amounts of both the carboxylated and decarboxylated forms, according to the Formula (1):

C_T_ = C_carb_ × 0.877 + C_dec_ (1)

where C_T_ was the total amount of a specific cannabinoid, and C_carb_ and C_dec_ were the amounts of its carboxylated and decarboxylated forms, respectively.

### 4.5. Statistical Analysis

Using the JMP software version 8.0 (SAS Institute Inc., Cary, NC, USA), the significant differences among the means were estimated through Tukey’s multiple test (*p* ≤ 0.05), whereas Pearson’s correlation coefficients were calculated to determine the existence and magnitude of the relationships (*p* ≤ 0.05) between all trait pairs.

Multivariate statistical analysis was performed using the online tool MetaboAnalyst version 5.0 [78]. The PCA was initially performed to reduce the complexity of the dataset with only a minimal loss of information. Then, an sPLS-DA was performed to optimize the segregation of the different treatments and identify the most discriminating traits. Before multivariate analyses, auto-scaling of data was carried out.

## 5. Conclusions

Hemp inflorescence extracts and purified compounds are emerging as interesting new ingredients in several industrial applications, including pharmaceuticals, nutraceuticals, functional foods, and cosmetics. In this context, CBD, for its potential health benefits, certainly represents the most valuable compound in hemp inflorescences, but the increased levels of phenolic compounds and tocopherols observed in the present study in CHT-treated plants may contribute to improving the beneficial properties of hemp inflorescence derivatives and increasing the economic value of industrial hemp production. Although the present study did not highlight striking differences between the different CHT treatments, interesting indications were obtained regarding the better response of the hemp inflorescences to the CHT 50 L treatment. However, these should be further investigated across different genotypes, monoecious and dioecious, as well as different environments and agronomical practices. Moreover, further study on the elucidation of mechanisms involved in the CHT-induced accumulation of specific phytochemicals in hemp inflorescences would enable better utilization of this biopolymer on this crop.

## Data Availability

All generated data are included in this article.

## Supplementary Materials

The following supporting information can be downloaded at: https://www.mdpi.com/article/10.3390/plants12213692/s1. Table S1. Content of individual secondary metabolites determined by HPLC, and total phenolic content (TPC), total flavonoid content (TFC), 2,2-azinobis-(3-ethylbenzothiazoline-6-sulphonic acid) (ABTS), and 2,2-diphenyl-1-picrylhydrazyl (DPPH) radical scavenging activities, determined spectrophotometrically in the inflorescences of hemp plants from the cv. Codimono exposed to different chitosan (CHT) treatments. Figure S1: Principal component analysis overview plot showing the five components. Figure S2: Sparse partial least squares discriminant analysis overview plot showing the five components.
