# Supplementary material for: Impact of Chitosan-Based Foliar Application on the Phytochemical Content and the Antioxidant Activity in Hemp (Cannabis sativa L.) Inflorescences
Source: Plants (Basel). 2023 Oct 26;12(21):3692. doi: 10.3390/plants12213692 (PMC10648115; doi:10.3390/plants12213692)
Supplement: Supplementary file 1 [file plants-12-03692-s001.zip › plants-2662879-supplementary.pdf]

Table S1. Content of individual secondary metabolites determined by HPLC, and total phenolic content (TPC), total flavonoid content (TFC), 2,2-azinobis-(3-ethylbenzothiazoline-6-sulphonic acid) (ABTS) and 2,2-diphenyl-1-picrylhydrazyl (DPPH) radical scavenging activities determined spectrophotometrically in the inflorescences of hemp plants from the cv. Codimono exposed to different chitosan (CHT) treatments.

	Treatment						
Trait[1]	**Ctr**	**CHT 50 L**	**CHT 50 M**	**CHT 50 H**	**CHT 250 L**	**CHT 250 M**	**CHT 250 H**
HPLC measurements							
Cannabinoids							
Cannabidiol	15223.75±2911.49	17568.37±1214.76	14920.04±158.70	14304.97±960.88	18249.72±2164.47	18625.78±388.43	16036.22±1225.16
Cannabigerol	393.78±125.64	461.01±84.51	372.03±14.92	382.49±97.50	663.44±156.41	496.09±93.22	488.09±102.27
Δ9-Tetrahydrocannabinol	377.72±103.72	474.60±38.02	383.15±11.90	354.33±54.73	460.75±75.01	447.00±17.41	412.33±43.10
Phenolic acids							
p-Hydroxybenzoic acid	84.77±7.39	98.30±4.33	72.61±7.14	86.45±8.30	88.69±3.30	69.67±3.57	75.06±5.90
Caffeic acid	20.29±0.76	22.42±0.53	23.10±2.38	18.86±2.29	21.89±1.57	21.05±1.25	29.30±0.44
Ferulic acid	422.19±32.74	368.74±22.39	404.23±37.30	436.11±17.30	353.38±29.43	353.18±35.42	474.27±17.69
p-Coumaric acid	89.34±4.11	102.15±3.28	102.38±0.88	96.97±2.43	95.63±1.14	86.29±5.64	104.37±0.59
Flavonoids							
Orientin	4531.23±53.44	5846.84±85.29	5910.07±107.46	5439.46±177.24	4357.32±182.50	5602.82±297.37	4404.35±83.52
Vitexin	4947.12±154.05	5926.49±95.20	5805.82±86.85	5300.64±57.76	4957.68±224.50	4538.75±325.87	5119.59±42.00
Isovitexin	1900.04±56.31	1746.42±76.00	2017.35±77.57	2041.12±86.85	1741.03±78.00	1814.54±56.14	2145.86±80.20
Luteolin	20.41±1.64	21.84±1.56	10.90±0.66	2.64±0.07	17.34±0.79	22.04±2.27	12.61±0.28
Apigenin	10.59±1.05	7.75±0.16	9.62±0.91	7.11±0.47	9.02±0.34	8.87±0.63	8.28±1.45
Epicatehin	280.36±27.70	389.35±2.63	356.32±16.44	278.69±36.97	244.32±17.06	363.94±25.65	398.06±12.21
Catechin	198.13±4.37	125.36±2.94	147.62±3.58	158.25±8.96	251.03±3.88	284.84±8.14	235.73±2.64

Carotenoids							
Lutein	318.92±36.87	292.67±17.32	284.67±13.14	321.42±9.68	274.08±13.49	229.08±18.29	277.42±5.75
β-Carotene	184.58±25.80	170.42±9.43	175.50±11.10	220.75±25.95	170.58±14.12	134.42±25.50	150.42±26.34
Tocopherols							
α-Tocopherol	416.26±9.81	730.48±36.27	691.35±10.51	667.48±10.58	642.30±24.90	603.25±13.44	682.34±14.88
β+γ-Tocopherol	47.45±3.42	86.59±7.10	62.88±1.28	73.99±1.90	69.69±1.37	77.81±5.18	78.97±6.10
δ-Tocopherol	10.17±0.30	12.27±1.29	17.93±1.38	13.92±1.37	11.02±0.28	15.89±0.44	11.96±1.60
Spectophotometric measurements							
TPC	42.23±0.42	64.88±3.28	71.58±2.25	57.28±1.00	59.00±1.88	67.44±2.15	58.79±1.56
TFC	16.52±0.39	21.05±0.71	18.55±0.34	16.75±0.31	20.71±0.10	17.86±0.59	17.68±0.32
Antioxidant activity							
ABTS	79.46±2.53	101.87±1.35	95.62±0.94	94.20±1.47	95.17±2.06	91.96±0.40	89.23±0.50
DPPH	115.27±2.78	137.21±4.05	120.87±2.55	129.68±2.20	135.89±1.46	125.56±3.76	139.00±0.95

[1]Data are reported as mean ± SE and are expressed as follows: secondary metabolites, μg/g dry weight (D.W.); TPC, mg ferulic acid equivalents/g D.W.; TFC, mg catechin equivalents/g D.W.; ABTS and DPPH radical scavenging activity, μmol Trolox equivalent/g D.W.

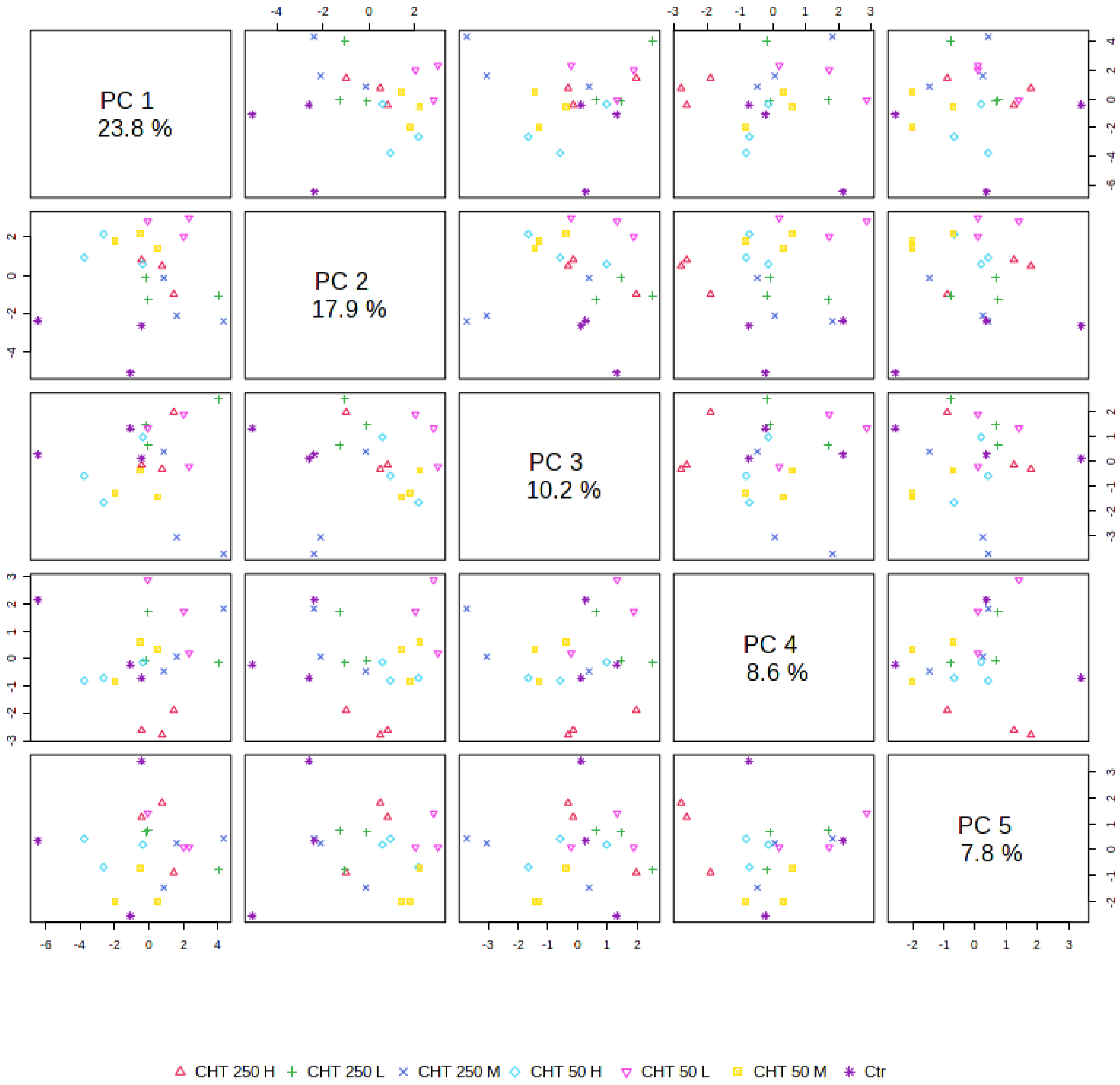

Figure S1. Principal component analysis overview plot showing the five components.

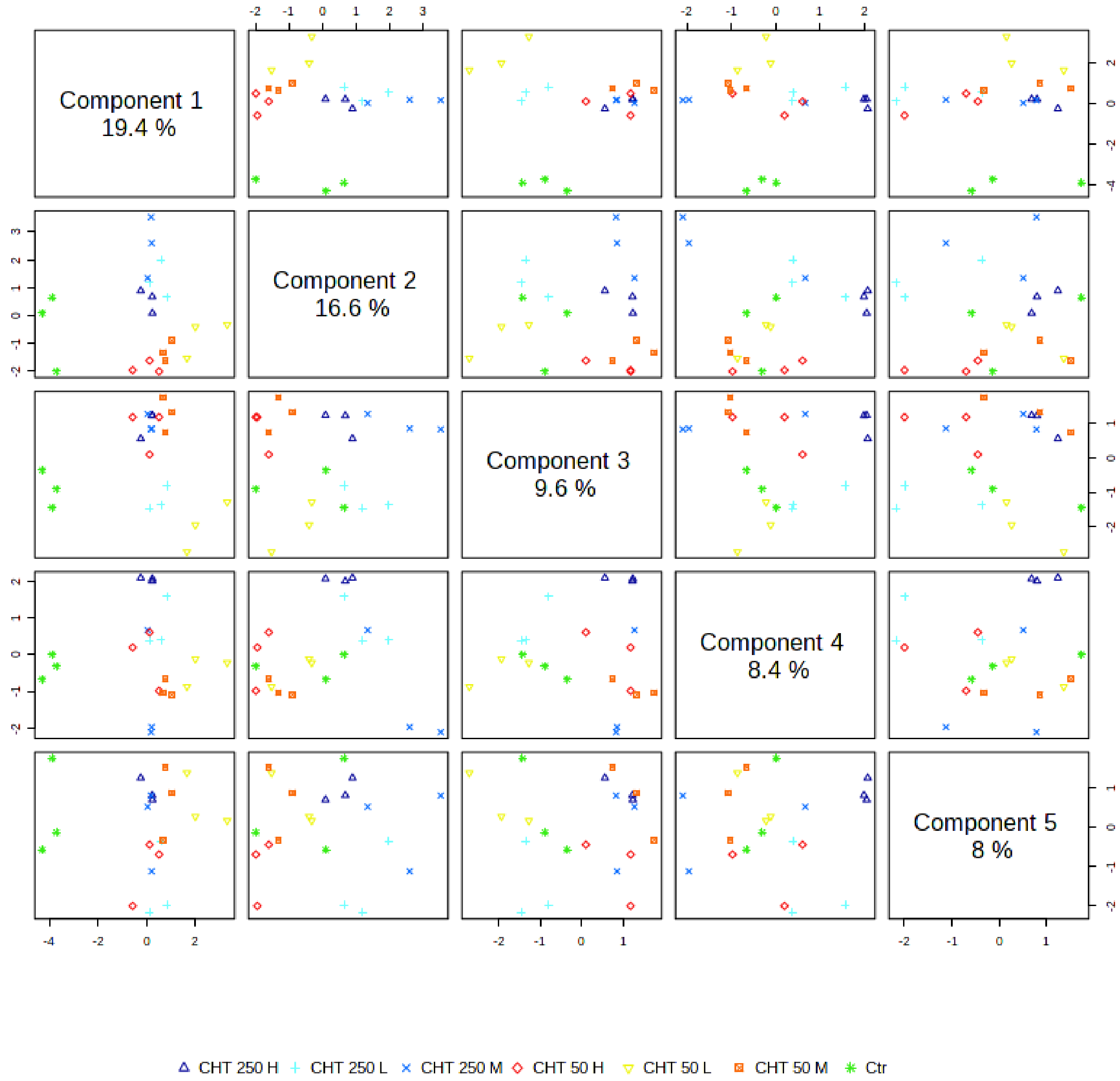

Figure S2. Sparse partial least squares discriminant analysis overview plot showing the five components.
